# Diagnosis of pulmonary tuberculosis via identification of core genes and pathways utilizing blood transcriptional signatures: a multicohort analysis

**DOI:** 10.1186/s12931-022-02035-4

**Published:** 2022-05-14

**Authors:** Qian Qiu, Anzhou Peng, Yanlin Zhao, Dongxin Liu, Chunfa Liu, Shi Qiu, Jinhong Xu, Hongguang Cheng, Wei Xiong, Yaokai Chen

**Affiliations:** 1 Division of Infectious Diseases, Chongqing Public Health Medical Center, Southwest University, Chongqing, China; 2 Department of Tuberculosis, Chongqing Public Health Medical Center, Southwest University, Chongqing, China; 3 National Center for Tuberculosis Control and Prevention, Chinese Center for Disease Control and Prevention, Beijing, China; 4 Department of Nutrition, The Seventh Medical Center of Chinese PLA General Hospital, Beijing, 100700 China; 5 Department of Oncology, Tongren People’s Hospital Affiliated to Guizhou Medical University, Tongren, China; 6 Faculty of Medicine, Kaifeng University, Kaifeng, China; 7 Department of Geriatrics, First Affiliated Hospital, Army Medical University, Chongqing, China

**Keywords:** Pulmonary tuberculosis, Hub genes, Expression profiling data, OAS1, IFIT1, IFIT3

## Abstract

**Background:**

Blood transcriptomics can be used for confirmation of tuberculosis diagnosis or sputumless triage, and a comparison of their practical diagnostic accuracy is needed to assess their usefulness. In this study, we investigated potential biomarkers to improve our understanding of the pathogenesis of active pulmonary tuberculosis (PTB) using bioinformatics methods.

**Methods:**

Differentially expressed genes (DEGs) were analyzed between PTB and healthy controls (HCs) based on two microarray datasets. Pathways and functional annotation of DEGs were identified and ten hub genes were selected. They were further analyzed and selected, then verified with an independent sample set. Finally, their diagnostic power was further evaluated between PTB and HCs or other diseases.

**Results:**

62 DEGs mostly related to type I IFN pathway, IFN-γ-mediated pathway, etc. in GO term and immune process, and especially RIG-I-like receptor pathway were acquired. Among them, *OAS1*, *IFIT1* and *IFIT3* were upregulated and were the main risk factors for predicting PTB, with adjusted risk ratios of 1.36, 3.10, and 1.32, respectively. These results further verified that peripheral blood mRNA expression levels of *OAS1*, *IFIT1* and *IFIT3* were significantly higher in PTB patients than HCs (all *P* < 0.01). The performance of a combination of these three genes (three-gene set) had exceeded that of all pairwise combinations of them in discriminating TB from HCs, with mean AUC reaching as high as 0.975 with a sensitivity of 94.4% and a specificity of 100%. The good discernibility capacity was evaluated d via 7 independent datasets with an AUC of 0.902, as well as mean sensitivity of 87.9% and mean specificity of 90.2%. In regards to discriminating PTB from other diseases (i.e., initially considered to be possible TB, but rejected in differential diagnosis), the three-gene set equally exhibited an overall strong ability to separate PTB from other diseases with an AUC of 0.999 (sensitivity: 99.0%; specificity: 100%) in the training set, and 0.974 with a sensitivity of 96.4% and a specificity of 98.6% in the test set.

**Conclusion:**

The described commonalities and unique signatures in the blood profiles of PTB and the other control samples have considerable implications for PTB biosignature design and future diagnosis, and provide insights into the biological processes underlying PTB.

**Supplementary Information:**

The online version contains supplementary material available at 10.1186/s12931-022-02035-4.

## Background

Tuberculosis (TB), together with HIV, is the primary cause of death around the world caused by an infectious agent. An estimated 10.0 million (range, 9.0–11.1 million) people fell ill with TB in 2018, resulting in 1.6 million deaths [1]. *Mycobacterium tuberculosis* (Mtb), the causative bacteria of TB, generally affects the lung and leads to pulmonary TB (PTB). This disease spreads when people with PTB expel Mtb into the air, including by coughing. Fatalities caused by TB are mostly preventable through a timely and accurate diagnosis [2]. Unfortunately, it is often neglected due to a lack of sensitive and expedient detection methods. Current detection methods, which largely rely on radiological assessments and checking for the presence of Mtb in patient samples, have many deficiencies. Current standard Mtb detection uses sputum cultures, which require 3 to 6 weeks to obtain results, thereby delaying the initiation of treatment. Molecular testing (e.g., GeneXpert), while allowing quicker diagnosis, also has limitations, including a high financial cost, inaccessibility in resource-poor settings, and relying on sputum availability to confirm the presence of bacteria by detecting pathogen DNA/RNA. Blood sample detection methods are more rapid, however, they lack high sensitivity and specificity [3]. To develop new biomarkers for TB diagnosis, it is necessary to enhance our understanding of TB pathogenesis and improve our knowledge of the regulatory network.

While the immunological response to Mtb is focused predominantly on the lungs, circulating immune cells in the peripheral blood determine its pathological status. Whole blood transcriptomic profiles serve as indispensable tools in ascertaining the molecular components underlying the infection and provide an insight into the host immune response in TB [4], especially the advancements of high-throughput sequencing and microarray technology have provided efficient tools for developing reliable diagnostic biomarkers [5]. The deposition of their datasets in public databases (such as the Gene Expression Omnibus (GEO)) offers possibilities for surveying molecular patterns from different perspectives via bioinformatics analysis [6]. Nonetheless, a single gene biomarker would have insufficient predictive power [7]. Studies have shown that gene signatures that include several genes are a better alternative [8, 9]. According to current information, studies on the multigene prognostic signatures of PTB are scarce. Transcriptome analyses of macrophages infected with either the virulent Mtb strain H37Rv (Rv) or the avirulent Mtb strain H37Ra (Ra) confirmed the gene expression of immune cells differs under Mtb-infection [10] but lacks diagnostic evaluation. Blischak et al. measured gene expression levels in Mtb-infected and non-infected dendritic cells to predict TB susceptibility [11], however, it was based on a small population (25 samples). Both are laboratory based experiments, and not directly based on patient data. Thus, the mechanisms and functions of mRNA in PTB require further study. As such, many efficient and sensitive mRNA signatures will need to be identified for PTB diagnosis.

Although a few studies have investigated host response to PTB infection using microarray-based whole-genome expression profiles in peripheral blood, the results have been inconsistent, or even contradictory [12–14]. However, one common finding across these studies is that despite the inconsistencies, the gene expression in PTB patients differs from that of healthy individuals. Thus, there is a need to recapitulate transcriptomic signatures in several studies worldwide using independent clinical cohorts, as well as in meta-analyses combining several of these cohorts for further analysis.

The latest study used meta-analysis to integrate transcriptome datasets from different studies and screen for TB biomarkers in patients who were HIV-positive [15]. In our study, we will analyse data for PTB while eliminating any potential interference from HIV by using HIV-negative patients only. In this study, whole blood gene expression datasets from two separate studies were selected and analyzed. Deregulated mRNAs were acquired and functionally annotated to explore the potential pathways in PTB. By integrating the information on gene expression and function, a protein–protein interaction (PPI) network was constructed to conduct modular analysis and immune process involvement. After which, hub genes with high degrees of connectivity were selected. Another independent dataset with PTB patients and healthy participants was then used to optimize hub gene selection according to their PTB diagnostic power, resulting in a three-gene set which was verified with an independent sample set. A receiver operating characteristic (ROC) curve was drawn to estimate the diagnostic power of this three-gene set between PTB and the healthy group from a series of datasets. Furthermore, a random forest (RF) classifier based on this three-gene set was also constructed to test their ability to discriminate PTB from other diseases (i.e., TB was considered in the differential diagnosis but then excluded). The information acquired provided insights into the immunotherapies and clinical diagnosis of PTB.

## Methods

### Microarray data

All of 11 datasets (GSE42834, GSE83456, GSE56153, GSE19491, GSE28623, GSE34608, GSE54992, GSE62525, GSE147964, GSE147690 and GSE37250) were retrieved from the GEO database (https://www.ncbi.nlm.nih.gov/geo/) based on the following criteria: (1) adult PTB patients: (i) culture-confirmed Mtb in either sputum or bronchoalveolar lavage; (ii) have not started TB treatment; (2) healthy controls (HCs) or other disease (i.e., TB was considered in the differential diagnosis but then excluded) controls: (i) matched age, gender and ethnicity to patients; (ii) negative interferon (IFN) -γ release assays (IGRAs). The exclusion criteria for the patients and healthy controls were the presence of other medical conditions (including any immunosuppression, such as HIV infection) and pregnancy. All data used are available online. No animal or human experiments were carried out by any of the authors involved in this study.

**Fig. 1:**
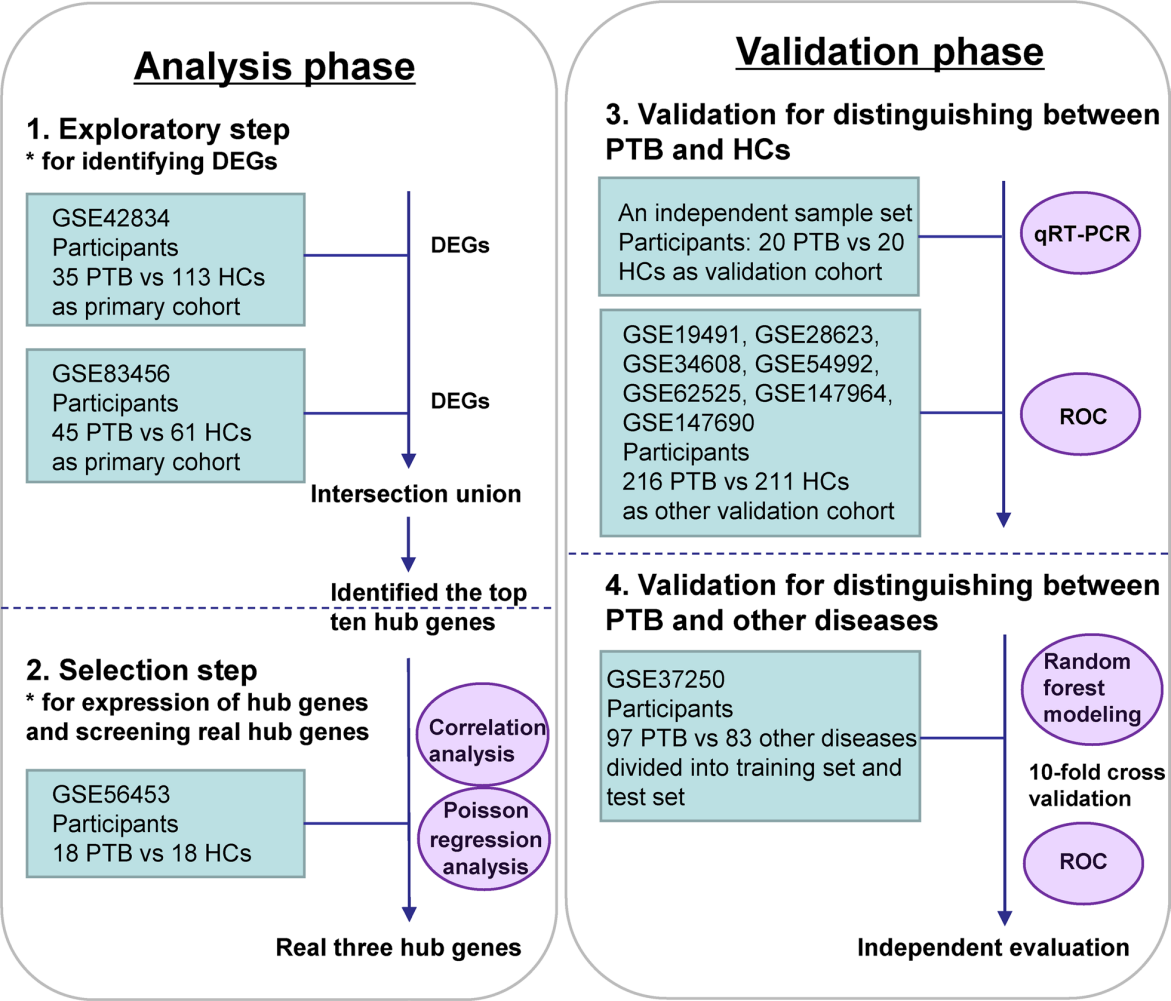
Flow diagram of procedure of data collection

The workflow of data collection is shown as Fig. 1. Briefly, GSE42834 (35 PTB samples and 113 HCs and GSE83456 (45 PTB samples and 61 HCs) were used to identify any differentially expressed genes (DEGs), obtaining ten hub genes. Then, GSE56153 (18 PTB and 18 HCs) was used to screen and evaluate the selected hub genes which were verified with an independent sample set (20 PTB and 20 HCs). To further evaluate the performance of our selected gene-set, its performance of differentiating PTB from HCs was evaluated using seven independent datasets (216 PTB and 211 HCs). In addition to the gene-set ability to distinguish between PTB and other diseases, GSE37250 (97 PTB and 83 other diseases) was used to train RF machine learning algorithm with tenfold cross validation, which was then used in the test set to evaluate its performance.

### Data processing of DEGs

GEO2R (https://www.ncbi.nlm.nih.gov/geo/geo2r/) was used to determine the differentially expressed genes (DEGs) between samples from PTB patients and healthy individuals, followed by the calculation of the false discovery rate (FDR) and log2 (fold change) (logFC). Genes that met the cutoff criteria (|logFC|≥ 1.5 and FDR < 0.05) were considered to be differentially expressed [16]. Each dataset underwent statistical analysis. Then, any intersecting parts were identified using the Venn diagram webtool (bioinformatics.psb.ugent.be/webtools/Venn/). SangerBox, a biological information visualization tool, was used to produce volcano plot and heatmap, and any genes that significantly changed (up and downregulated genes) were presented (|logFC|≥ 1.5 and FDR < 0.05).

### GO, KEGG pathway and GSEA analysis

Gene Ontology (GO) analysis is a valuable method commonly used for large-scale functional enrichment research [17]. Gene functions are usually categorized into cellular component (CC), molecular function (MF), and biological process (BP). Kyoto Encyclopedia of Genes and Genomes (KEGG) is a common database resource that lists large amounts of drugs, chemicals, diseases, biological pathways, and genome data [18]. The DEGs were subjected to GO annotation and KEGG pathway enrichment analysis utilizing the Database for Annotation, Visualization, and Integrated Discovery (DAVID) tools (DAVID Bioinformatics Resources 6.8) [19]. Statistical significance was achieved when gene counts ≥ 3 and *P* < 0.05, whereas *P* < 0.05 for GO analysis of the top ten hub genes with DAVID. Gene Set Enrichment Analysis (GSEA) was conducted using GSEA v4.1.0 software (http://www.gsea-msigdb.org/gsea/index.jsp) and certain gene sets used in the present study for GO and KEGG terms were downloaded from the Molecular Signatures Database (MSigDB, http://software.broadinstitute.org/gsea/msigdb/index.jsp, v7.2) [20]. Go and KEGG signaling pathway analysis were performed through pre-ranked GSEA. Members of a gene set were pre-ranked based on their *P*-value with the up-regulated gene at the top and the down-regulated genes at the bottom. Gene sets were considered to be significantly enriched by a combined criteria of |normalized Enrichment Scores (NES)|> 1, *P* < 0.05 and FDR < 0.25. PPI network construction and hub gene identification.

The Search Tool for the Retrieval of Interacting Genes (STRING) database was used for PPI information analysis. Previously identified DEGs were compared to the STRING database to evaluate any potential PPI relationship. PPI pairs with a combined score of ≥ 0.7 were extracted. The PPI network was subsequently clearly shown with Cytoscape 3.7.1, equipped with ClueGo and CluePedia plugins [21]. Furthermore, modules of the PPI network were verified by Molecular Complex Detection (MCODE) in Cytoscape with the following standard: degree cutoff = 2, max. depth = 100, k-core = 2, and node score cutoff = 0.2. In addition, Interrelation analysis in immune system process was performed and then visualized using ClueGO + CluePedia plugins. Finally, CytoHubba, a Cytoscape plugin, was performed for the calculation of each protein node degree [22]. In this study, 10 highest-ranking genes (as defined by their degree) were identified as hub genes. The PPI networks were then used to verify these hub genes, using the maximal clique centrality (MCC) and maximum neighborhood component (MNC) algorithms of cytoHubba [23].

### Evaluation of ten hub genes and their evaluation in PTB and HC samples

For the GEO database, a link is provided at the bottom of the page to the series matrix file(s), which contains the expression values for each gene (probe set) and microarray. The values have been normalized using the normexp method and quantile normalized between arrays, rendering the samples cross-comparable. R scripting was used to extract the expression values of a small number of genes (probe sets) of interest and the clinical data from the data matrices downloaded from GEO. Log2 transformation on values was performed, which represented the expression levels of the genes for further analysis. The other independent GEO datasets were used to further evaluate the ten hub genes. Briefly, we used GSE56153 dataset to compare the indicated gene expression in PTB patients versus HCs. Besides, correlation analysis and multivariate poisson regression analysis were used to estimate the relationship between the ten hub genes and PTB variables. Furthermore, we used seven independent datasets (GSE19491, GSE28623, GSE34608, GSE54992, GSE62525, GSE147964, GSE147690) to evaluate the three-gene set (*OAS1*, *IFIT1* and *IFIT3*) optimized for diagnostic power from the ten hub genes obtained above. Additionally, ROC analysis was used to determine this three-gene set’s ability to distinguish PTB patients from HCs, and the overall accuracy was revealed by determining the area under the curve (AUC) in terms of sensitivity and specificity.

### Evaluation of the three-gene set in an independent sample set

We further evaluated the three genes (*OAS1*, *IFIT1* and *IFIT3*) using peripheral blood samples from an independent sample set, including 20 patients with pulmonary tuberculosis and 20 healthy volunteers that were matched according to age and sex. Active PTB patients were recruited from Chongqing Public Health Medical Center between April 2021 and June 2021. All PTB patients were recruited with typical clinical symptoms, chest radiograph revealing TB lesion, at least 2 consecutive positive sputum smears or a positive sputum culture, or a positive Xpert MTB/RIF result. They had not received anti-TB treatment within the past 30 days. Healthy controls were recruited from regular physical examination campaigns, which were conducted in Chongqing between March 2021 and June 2021. Healthy controls were people with a normal chest radiograph and no clinical symptoms of diseases. Individuals with positive human immunodeficiency virus (HIV), positive hepatitis B virus (HBV) or hepatitis C virus (HCV), diabetes, malignancies, severe autoimmune diseases, and those who took immunosuppressive or immunopotentiator agents, or were in pregnancy or lactation were excluded. The demographic characteristics of all 40 participants in this study are shown in an additional word file in more detail (see in Additional file 1). This study was performed in accordance with the guidelines of the Helsinki Declaration and was approved by the Ethics Committee of the Chongqing Public Health Medical Center (No. 2021-018-01-KY). Written informed consents were obtained from each participant before blood sample collection.

### PBMCs isolation and quantitative real-time PCR analysis

Peripheral blood samples (5 ml) from participants were collected in heparin-containing vacutainer tubes from each subject. Peripheral blood mononuclear cells (PBMCs) were separated by density gradient using Lymphocyte Cell Separation Media (Tianjin Haoyang Biological Manufacture Co., Ltd., China) within 6 h of blood collection. Total RNA was extracted from PBMCs using TRIzol reagent (Invitrogen, MA, USA) according to the manufacturer’s protocol. A total of 1.5 μg of purified RNA was reverse transcribed to cDNA using Goldenstar RT6 cDNA Synthesis Kit Ver 2 (TSK302S, Tsingke Biotechnology Co., Ltd., China) according to the manufacturer’s instructions. SYBR Green (2 × T5 Fast qPCR Mix (SYBR Green I, Tsingke Biotechnology Co., Ltd., China) uptake in double-stranded DNA was measured using ABI 7900 Real-time PCR System (Applied Biosystems, Inc., USA). 2-ΔΔCT was calculated and used to determine relative gene expression. The reference gene was GAPDH. The primer sequences of the target genes in the qPCR analysis are shown in an additional word file in more detail (see in Additional file 2).

### Predictive performance of the three-gene set in distinguishing PTB from other diseases

To further quantitatively determine the performance of the three-gene set in predictively classifying PTB and other diseases, we used the independent dataset GSE37250 containing 97 PTB patients and 83 patients with other diseases to construct the multiple regression model as previously described [24, 25]. According to the description of the study, the data were obtained from[12], after intensive investigation, any case with a confirmed alternative diagnosis to TB, no microbiological evidence of TB, or an absence of TB symptoms at the time of follow-up all while showing observed improvement of clinical symptoms to non TB treatment, was recruited as an other disease case. RF machine learning algorithms were used to train the regression model [26–28]. In this method, the reliability of the result was judged, using the tenfold cross validation. Briefly, RF predictive model was all iteratively trained and tested using tenfold cross validation with early stopping mechanisms to prevent overfitting. In this validation paradigm, data were partitioned into ten random segments or folds. Training occurred on nine of the folds as the training set, and the remaining fold as the test set was used to monitor performance for overfitting. Each of the ten models trained and was then tested on the test set partitioned prior to hyperparameter tuning, and the final metrics reported were averages for the metrics across the ten models. The classification performances of RF with a three-gene set were evaluated using AUC. Simultaneously, accuracy, sensitivity, and specificity were calculated using the confusion matrix of classification results.

### Statistical analysis

Statistical analysis was performed using SPSS 26.0 (SPSS Inc., USA) and GraphPad Prism 8.0.2 (GraphPad, USA). Data were analyzed using one-way analysis of variance (ANOVA) to compare multiple groups, or Student’s *t*-tests (two-sided) to compare two groups in condition of homogeneity of variance, otherwise, the Mann–Whitney U test was used. Correlations between the ten hub genes and PTB variable were analyzed using point-biserial correlation tests, then a quantitative relationship of the interdependence of those was analyzed using multivariate poisson regression analysis with a robust variance estimate. R software (version 4.0.0; R Foundation for Statistical Computing) was used for bioinformatics analysis. RF predictive model was implemented in R using the package for randomForest and caret respectively. The importance of the variable was measured in terms of the average normalized mean square error decline caused by the deletion of the variable, the larger the value, the more important for the variable. The prediction capacity of variables was evaluated by the ROC curve and AUC value using the ROCR package in R. Data are shown as the mean ± standard deviation (SD). *P* < 0.05 indicated a statistically significant difference.

## Results

### Identification of DEGs

**Fig. 2:**
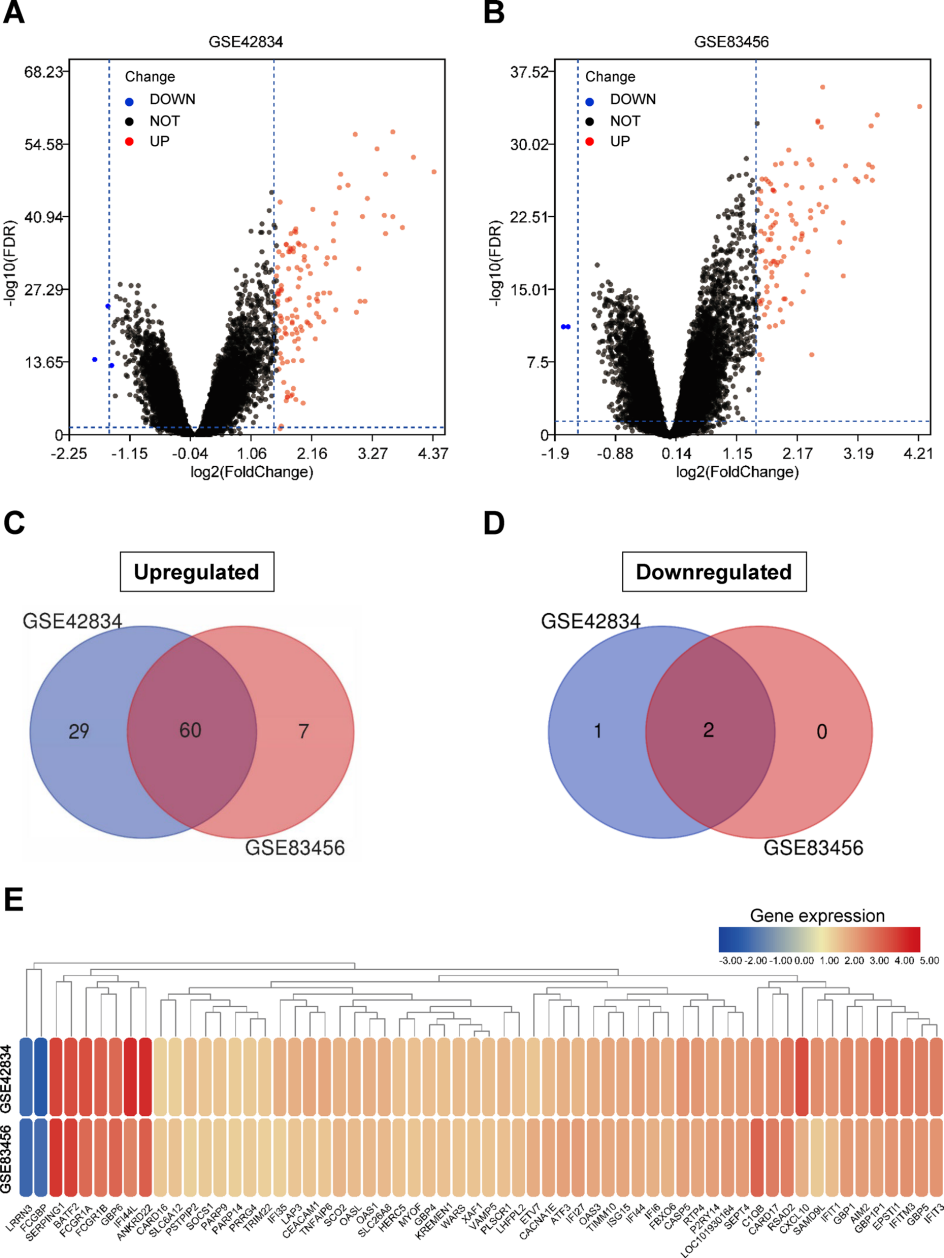
All genes and DEGs common in the two GEO datasets. **A** Volcano plot of all genes in GSE42834 gene chip. Red and green dots denote upregulated and downregulated genes, respectively. Black dots show the remaining genes without significant changes in expression. **B** Volcano plot of all genes in GSE83456 gene chip. **C** Venn diagram of upregulated genes common in the two GEO datasets. **D** Venn diagram of downregulated genes common in the two GEO datasets. **E** Heatmap of DEGs in the two GEO datasets (60 upregulated and 2 downregulated genes). Legend represents gene expression. Red and blue colors denote upregulation and downregulation, respectively

Two gene expression profiles (GSE42834 and GSE83456) were chosen for this study. GSE42834 included 35 PTB samples (PTB group) and 113 healthy control samples (HC group), while GSE83456 included 45 PTB samples (PTB group) and 61 HC samples (HC group) (Table 1). DEG identification was performed by comparing PTB and HC samples. At FDR < 0.05 and |logFC|≥ 1.5, 92 DEGs (89 upregulated and 3 downregulated) were found in GSE42834. In GSE83456, 69 DEGs (67 upregulated and 2 downregulated) were determined. All genes were plotted in Fig. 2A, B. Subsequently, intersections between DEG profiles (Fig. 2C, D) were observed using Venn analysis. Sixty-two DEGs were differentially and significantly expressed in the two groups. Among these DEGs, 60 were notably upregulated, while 2 were downregulated. The expression levels of these genes were demonstrated. The logFC of DEGs in the two GEO datasets is shown in the heatmap, appearing well clustered (Fig. 2E).

**Table 1:** Statistics of the two microarray databases derived from the GEO database

Dataset ID	PTB	HC	Total number
GSE42834	35	113	148
GSE83456	45	61	106

GEO: Gene Expression Omnibus; PTB: pulmonary tuberculosis; HC: healthy control

### Functional enrichment analyses of DEGs

**Fig. 3:**
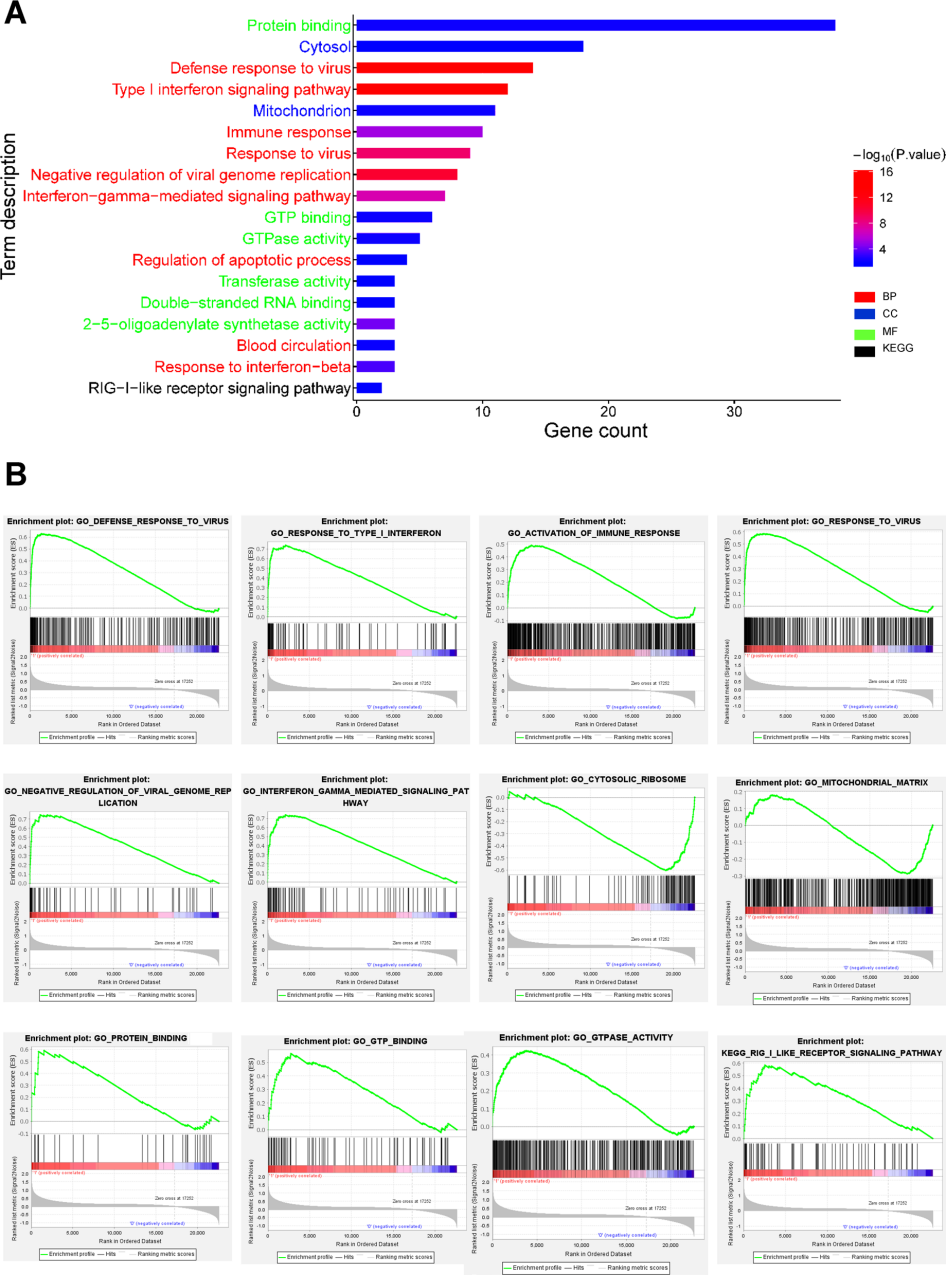
**A** Significantly enriched GO terms and KEGG pathways of DEGs with *P* < 0.05 and gene counts ≥ 3. Font colors of Y-axis label correspond to different enriched terms (BP, CC, MF terms of GO or KEGG pathways) of DEGs. Legend indicates the significance of the term (−log10 *P*-value). **B** Results of Gene Set Enrichment Analysis (GSEA) in GO and KEGG terms showed differential enrichment of genes in PTB and HC groups. “1” represents PTB, “0” represents HCs

To evaluate the biological role of DEGs in PTB disease, GO function enrichment, KEGG pathway, and GSEA analysis were performed. As shown in Fig. 3A and an additional word file in more detail (see in Additional file 3), GO analysis indicated that the DEGs were mainly enriched in BPs, including defense response to virus, type I IFN signaling pathway, immune response, response to virus, negative regulation of viral genome replication, IFN-γ-mediated signaling pathway, regulation of apoptotic process, response to IFN-β, and blood circulation. MF analysis showed that the DEGs were considerably enriched in protein binding, GTP binding, GTPase activity, 2′-5′-oligoadenylate synthetase (OAS) activity, double-stranded RNA binding, and transferase activity. In CC, DEGs were enriched in the cytosol and mitochondrion. Furthermore, DEGs largely enriched in RIG-I-like receptor (RLR) signaling pathway were identified using KEGG pathway analysis. Finally, GSEA analysis in GO and KEGG terms verified that the results were positively correlated with PTB (Fig. 3B), implying that DEGs in PTB patients are potentially involved in regulation of innate and adaptive immune responses.

### PPI network construction and modular analysis

**Fig.4:**
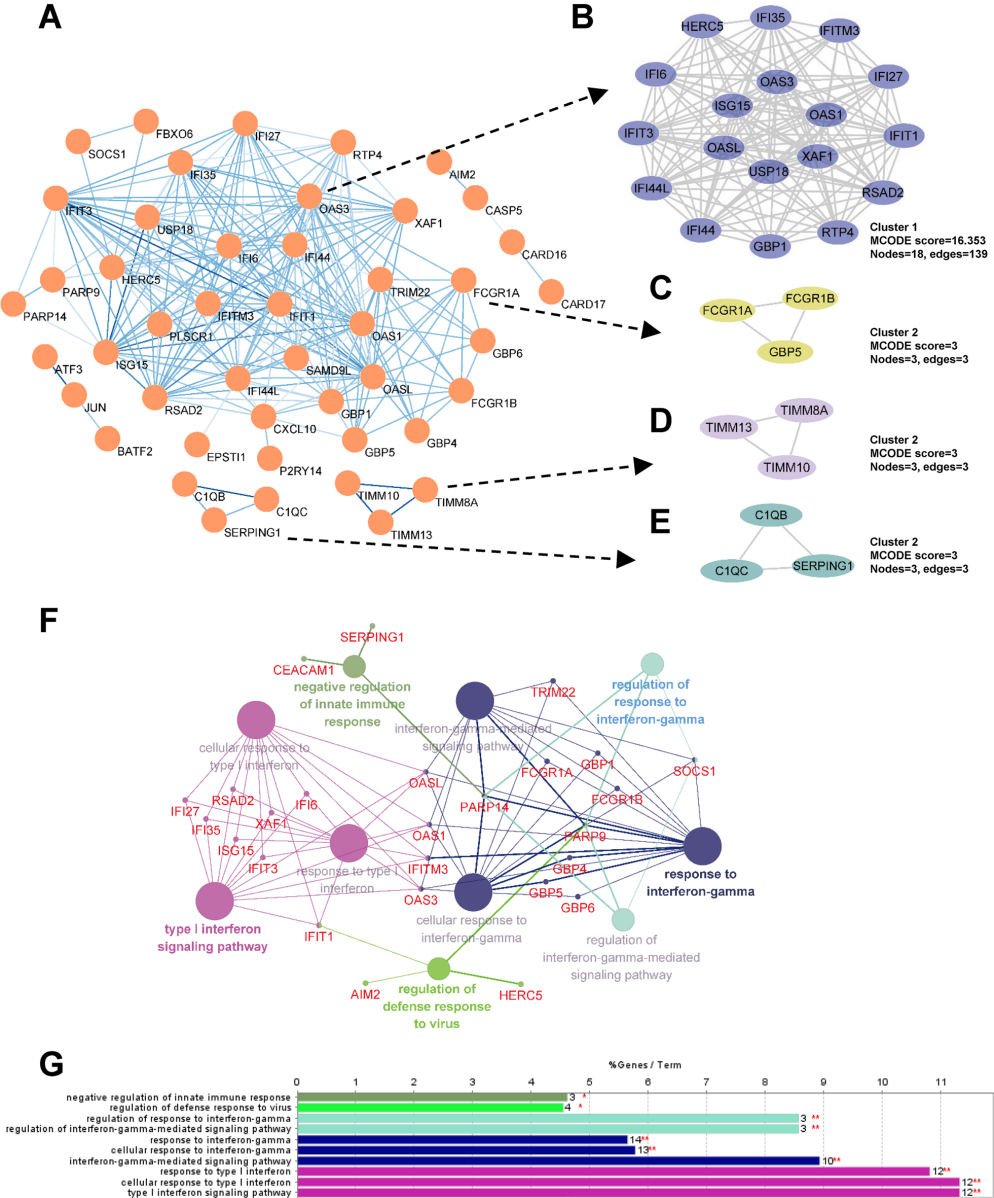
PPI network of DEGs and module analysis. **A** DEG PPI network constructed by STRING online database. Circular nodes represent proteins. Edges indicate the interaction between two proteins. The color intensity of the nodes or edges is based on the degree or combined score in the DEGs. **B–E** Module analysis via Cytoscape software (degree cut-off = 2, node score cut-off = 0.2, k-core = 2, and max. depth = 100) and four central modules were built based on the PPI network. **F** Interrelation analysis between immune system pathways. All genes from all pathways were noted in red. **G** Count number of genes involved in the identified pathways

All DEGs were submitted to STRING in order to construct the PPI network visualized using Cytoscape. As shown in Fig. 4A, 204 edges and 60 nodes were recognized (PPI enrichment, *P* < 1.0e−16). MCODE plugin was then used to select the top four central modules in the PPI network. Module 1 had an MCODE score of 16.353 and consisted of 18 nodes with 139 edges. The rest had a score of 3 and consisted of 3 nodes with 3 edges (Fig. 4B–E). Furthermore, interrelation analysis in immune system pathways was performed using ClueGO + CluePedia plugins. All of the DEGs were found to be largely enriched in terms related to type I IFN signaling pathway, IFN-γ-mediated signaling pathway, regulation of defense response to virus, and negative regulation of innate immune response (Fig. 4F, G). Notably, most genes from module 1 and module 2 were involved in those immune processes.

### Identification and analysis of hub genes

The top ten hub genes were identified and evaluated with regard to the degree of connectivity in the PPI network (Table 2). The results demonstrated that the *2′-5′-OAS1* gene had the highest connectivity degree (degree = 26), followed by the *OAS3* (degree = 25), 2′-5′-OAS-like (*OASL*; degree = 24), IFN-induced protein with tetratricopeptide repeats 3 (*IFIT3*; degree = 22), IFN-induced protein 44-like (*IFI44L*; degree = 21), IFN-induced protein 44 (*IFI44*; degree = 20), IFN-stimulated gene 15 (*ISG15*; degree = 20), radical S-adenosyl methionine domain containing 2 (*RSAD2*; degree = 20), IFN-induced protein with tetratricopeptide repeats 1 (*IFIT1*; degree = 20), and XIAP-associated factor 1(*XAF1*; degree = 19). We used another MCC and MNC algorithm of cytoHubba to create the PPI network and found that the top ten hub genes were common in the three algorithm methods (Table 2). All top ten hub genes were contained in aforementioned module 1, indicating many of which correspond to known molecular complexes in densely connected regions of PPI networks.

**Table 2:** Top ten hub genes with a high connectivity degree ranked by three different algorithms

Gene symbol	Gene description	Degree	MCC	MNC
*OAS1*	2'-5'-Oligoadenylate synthetase 1	26	1.88E+10	26
*OAS3*	2'-5'-Oligoadenylate synthetase 3	25	1.88E+10	25
*OASL*	2'-5'-Oligoadenylate synthetase like	24	1.88E+10	24
*IFIT3*	Interferon induced protein with tetratricopeptide repeats 3	22	1.88E+10	22
*IFI44L*	Interferon induced protein 44 like	21	1.87E+10	20
*IFI44*	Interferon-induced protein 44	20	1.87E+10	20
*ISG15*	Interferon stimulated gene 15	20	1.88E+10	20
*RSAD2*	Radical S-adenosyl methionine domain containing 2	20	1.88E+10	20
*IFIT1*	Interferon induced protein with tetratricopeptide repeats 1	20	1.88E+10	20
*XAF1*	XIAP-associated factor 1	19	1.88E+10	19

MCC: maximal clique centrality; MNC: maximum neighbourhood component

**Fig. 5:**
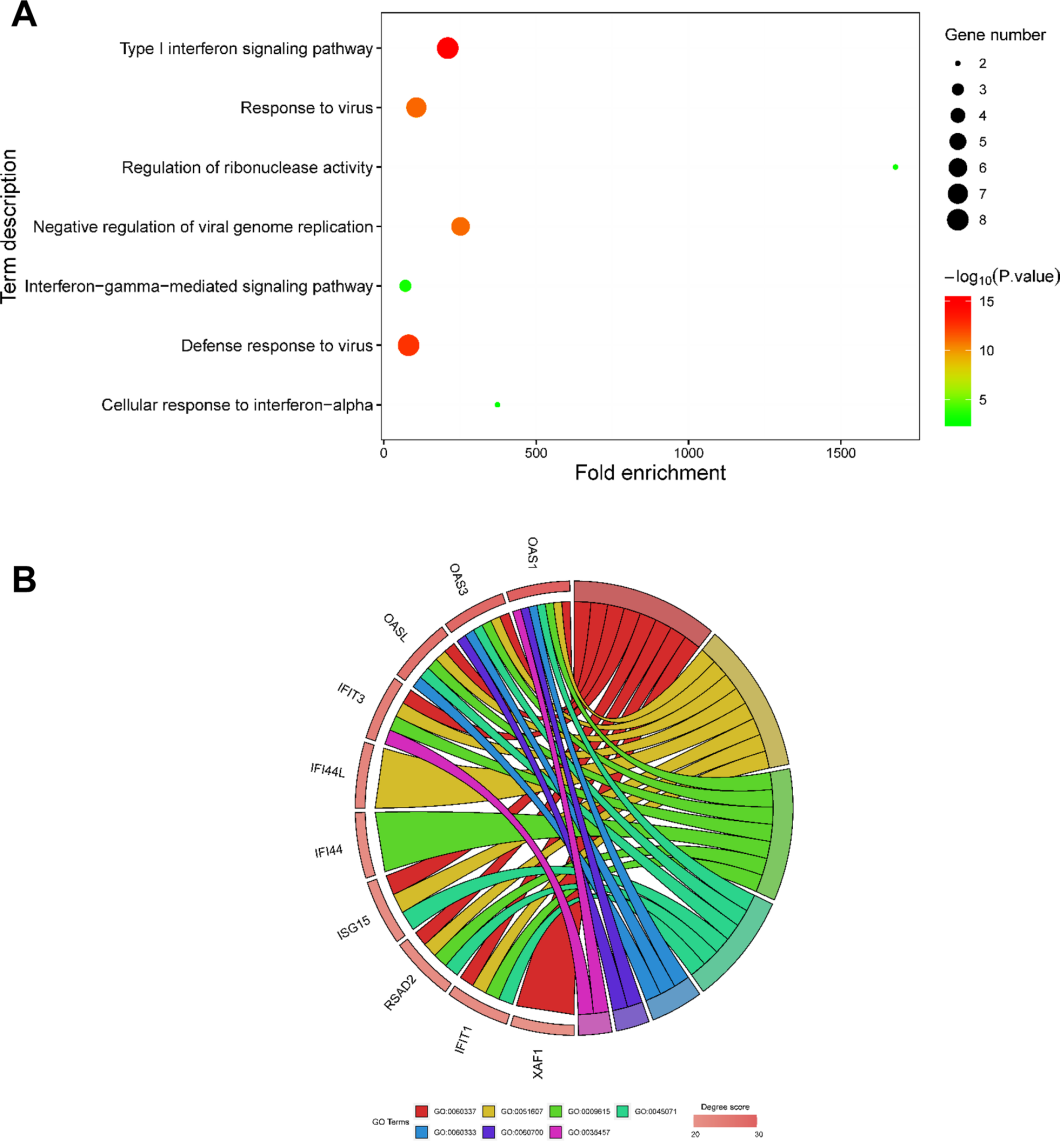
The GO enrichment results of the top ten hub genes. The bubble plot and chord diagram show the GO term (BP) (**A**) related to the top ten hub genes (**B**)

For a better understanding of the potential biological functions of the top ten hub genes, we used DAVID to re-analyze BP enrichment (*P* < 0.05) (Fig. 5). The results showed that these hub genes were functionally associated with several critical biological processes consistent with the DEGs, including type I IFN signaling pathway, defense response to virus, response to virus, negative regulation of viral genome replication, IFN-γ-mediated signaling pathway (Fig. 5A, B), predicting the hub ten genes might be really representative of many DEGs.

### Evaluation of the hub genes

**Fig. 6:**
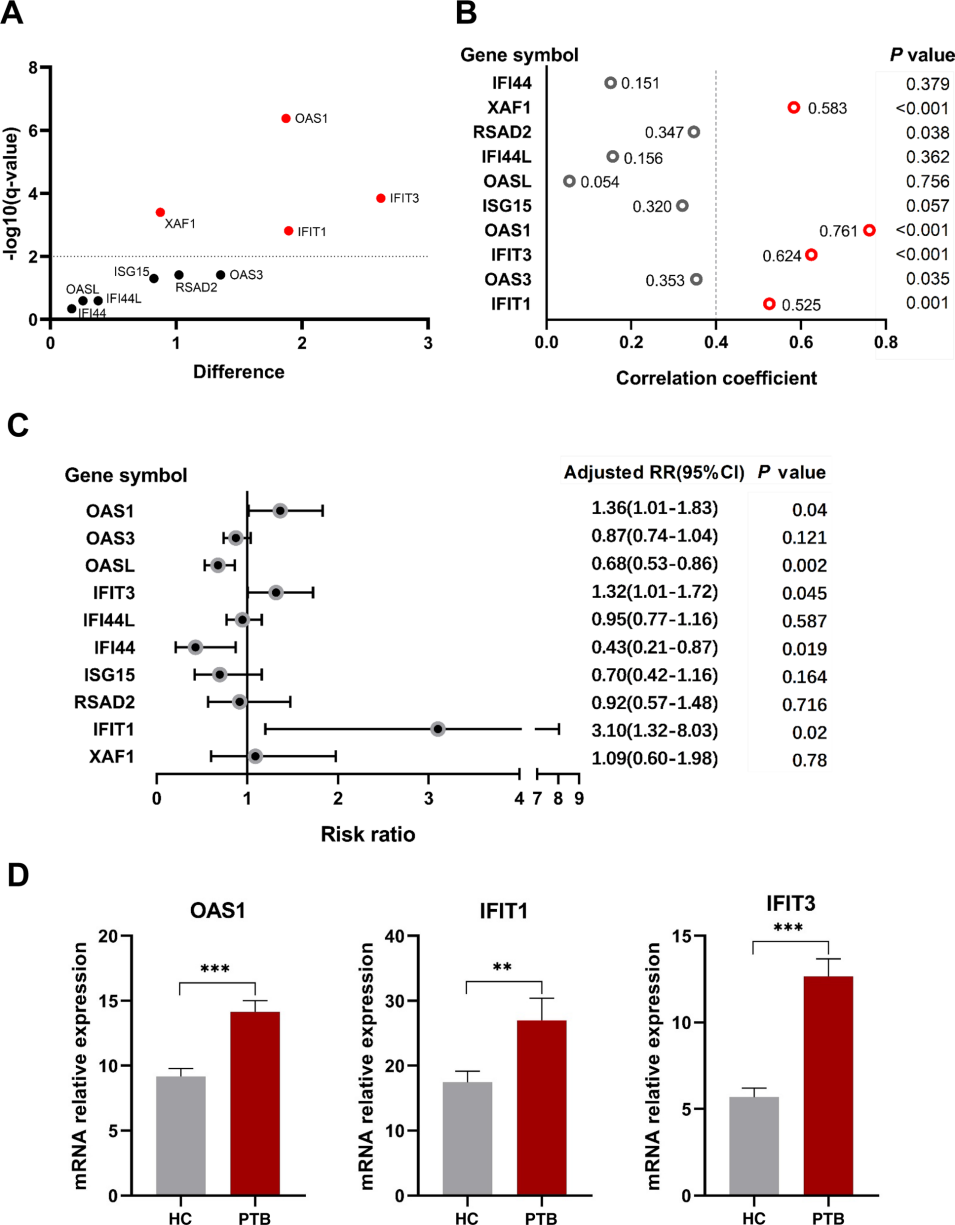
Expression of the ten hub genes and screening in an independent dataset (GSE56153). **A** Scatter plot from multiple *t* tests. mRNA expression levels of *ISG15, OAS1, IFIT3, OAS3, IFIT1, OASL, IFI44L, RSAD2, XAF1* and *IFI44* in the peripheral blood from PTB patients were compared with those in HC groups. Data were analyzed with multiple *t* tests and the scatter plot is created via GraphPad Prism 8.0.2. Each dot represents one gene and red dots denote genes with significant changes in expression (*q* < 0.01). The X axis is the difference between means for each gene from PTB and HC groups. The Y value plots the minus logarithm of the *q*-value. A dotted grid line is shown at Y = −log10(0.01). **B** Correlation of *ISG15, OAS1, IFIT3, OAS3, IFIT1, OASL, IFI44L, RSAD2, XAF1* and *IFI44* expression levels and PTB variable. Correlations were analyzed using point-biserial correlation tests and correlation coefficients of these genes were plotted. Red circles denote genes with correlation coefficient > 0.5 and *P* < 0.01. **C** Forest plot of the association between the ten hub genes and PTB in GSE56153 dataset. These ten hub genes from PTB patients and HCs were analyzed using multivariate poisson regression analysis with robust variance estimate. Metaanalysis of those was conducted, and adjusted RR, 95% CI of each gene and corresponding *P* value were calculated and plotted in the forest plot. **D** mRNA expression values of the three genes (*OAS1*, *IFIT1* and *IFIT3*) in PTB and HC from an independent sample set by qRT-PCR. The mRNA values of the evaluated genes were normalized to the housekeeping gene GAPDH. The numbers of participants in validation test were the following: PTB, n = 20; HC, n = 20. **P < 0.01, ***P < 0.001

The other independent GEO datasets were used to further evaluate the expression of the hub genes. The RNA levels of the ten hub genes were evaluated using GSE56153, which revealed that 4 genes were statistically upregulated in the peripheral blood from PTB patients compared with HCs, namely *OAS1*, *IFIT1*, *IFIT3,* and *XAF1* (*q* < 0.01; Fig. 6A). Furthermore, the correlations between different expression levels of the ten hub genes and the PTB variable were presented in Fig. 6B. *OAS1*, *IFIT1*, *IFIT3,* or *XAF1 gene* was positively associated with PTB with correlation coefficients of 0.525 ~ 0.761 (all *P* < 0.01), suggesting these genes might be appreciated as the candidate indicators of PTB. Finally, multivariable poisson regression analysis indicated that *OAS1*, *IFIT1*, *IFIT3, OASL* and *IFI44* were included in the equation (all of *P* < 0.01), whereas adjusted risk ratios (RRs) of *OAS1*, *IFIT1* and *IFIT3* were 1.36 (95% CI 1.01–1.83; *P* = 0.04), 3.10 (95% confidence interval (CI) 1.32–8.03; *P* = 0.02) and 1.32 (95% CI 1.01–1.72; *P* = 0.045), respectively, in discriminating TB from the HC group (Fig. 6C). Taken together, these results revealed that *OAS1*, *IFIT1* and *IFIT3* as factors could indicate the risk of PTB disease, and the three-gene set (*OAS1*, *IFIT1* and *IFIT3*) might potentially be multi-combined diagnostic biomarkers in differentiating PTB and healthy groups.

We then sought to verify the three genes (*OAS1*, *IFIT1* and *IFIT3*) in an independent sample set and found that gene expression levels of *OAS1*, *IFIT1* and *IFIT3* in peripheral blood were statistically significant (all *P* < 0.01) higher in PTB patients than in healthy controls (Fig. 6D).

### Efficacy evaluation for the three-gene set predicting PTB versus HCs

**Fig. 7:**
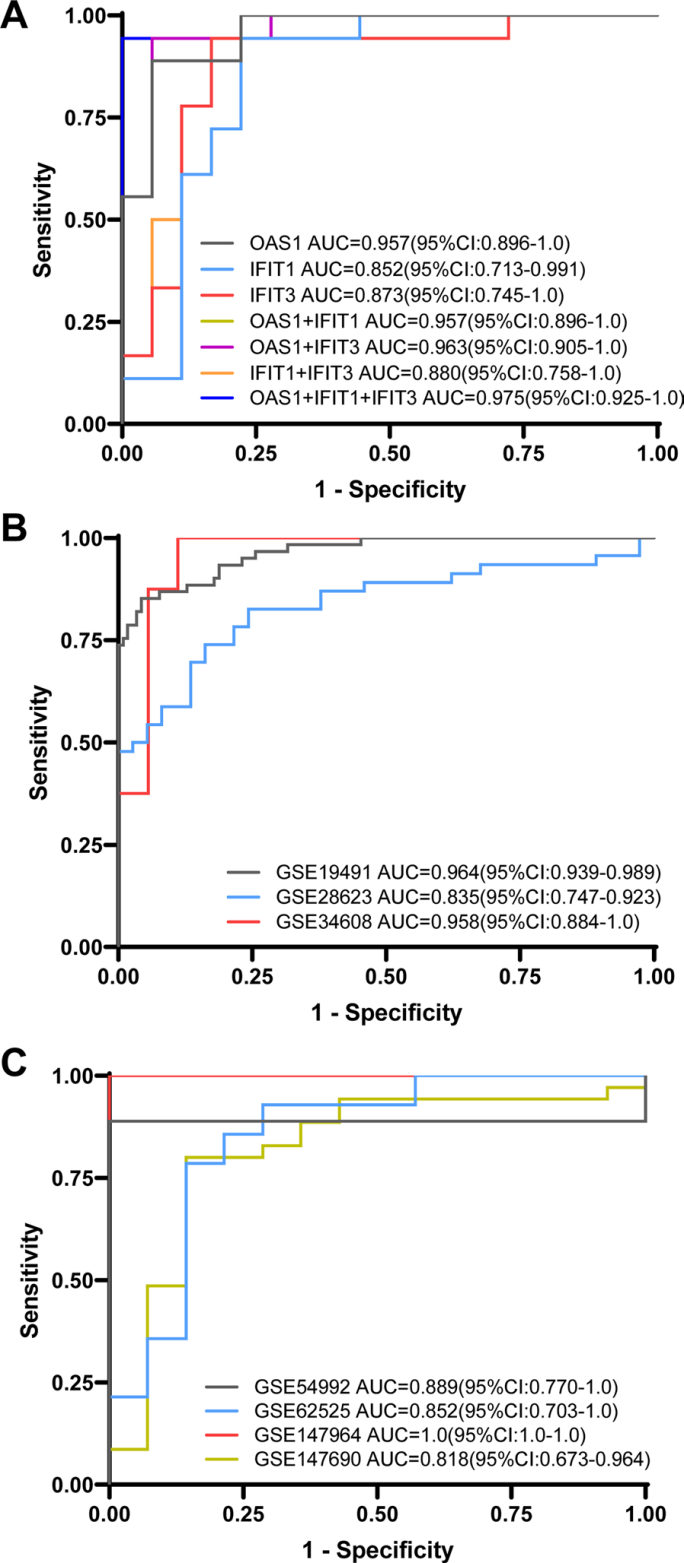
Efficacy evaluation for the three-gene set predicting PTB versus HCs by receiver operating characteristic curve (ROC) analysis. **A** ROC of the single gene or optional combinations of the three-gene set for discriminating PTB from HCs in independent GSE56153 dataset. 95% confidence interval of AUC was shown in the legend area, and the different colors presented different genes or combinations. **B**, **C** ROC of the three-gene set in the other seven independent datasets (GSE19491, GSE28623, GSE34608, GSE54992, GSE62525, GSE147964, GSE147690). 95% CI of AUC was shown in the legend area, and the different colors presented different datasets

To further detect whether these three genes (*OAS1*, *IFIT1* and *IFIT3*) or a combination thereof could discriminate TB from HCs, ROC analysis was performed to evaluate their discriminative potential in the validation sets. In GSE56153, as a result, the AUCs of *OAS1*, *IFIT1* and *IFIT3* were found to be 0.957 (95% CI 0.896–1.000), 0.852 (95% CI 0.713–0.991) and 0.873 (95% CI 0.745–1.000), respectively, in discriminating TB from HCs. However, The AUC values of the combination of all three genes could reach as high as 0.975 with a sensitivity of 94.4% and a specificity of 100%, which exceeded that of a combination of any two (Fig. 7A). In addition, seven independent datasets (GSE19491, GSE28623, GSE34608, GSE54992, GSE62525, GSE147964 and GSE147690) were used to compare HCs with PTB patients; these datasets contained a total of 211 HCs and 216 PTB patients (Table 3). As expected, in the validation datasets, the three-gene set distinguished PTB from HCs with a mean AUC of 0.902 (range 0.818–1), as well as mean sensitivity of 87.9% and a mean specificity of 90.2% (Fig. 7B, C). These results demonstrated that a better discriminative capacity between healthy individuals and those with PTB can be achieved via the combination of genes compared to any single gene.

**Table 3:** Statistics of the nine microarray databases derived from the GEO database

Dataset ID	PTB	HC	Other diseases	Total number
GSE56153	18	18	–	36
GSE19491	117	61	–	178
GSE28623	37	46	–	83
GSE34608	18	8	–	26
GSE54992	6	27	–	33
GSE62525	14	14	–	28
GSE147964	10	20	–	30
GSE147690	14	35	–	49
GSE37250	97	–	83	180

GEO: Gene Expression Omnibus; PTB: pulmonary tuberculosis; HC: healthy control; Other diseases: TB was considered in the differential diagnosis but then excluded

### Prediction accuracy of the three-gene set distinguishing PTB from other diseases

**Fig. 8:**
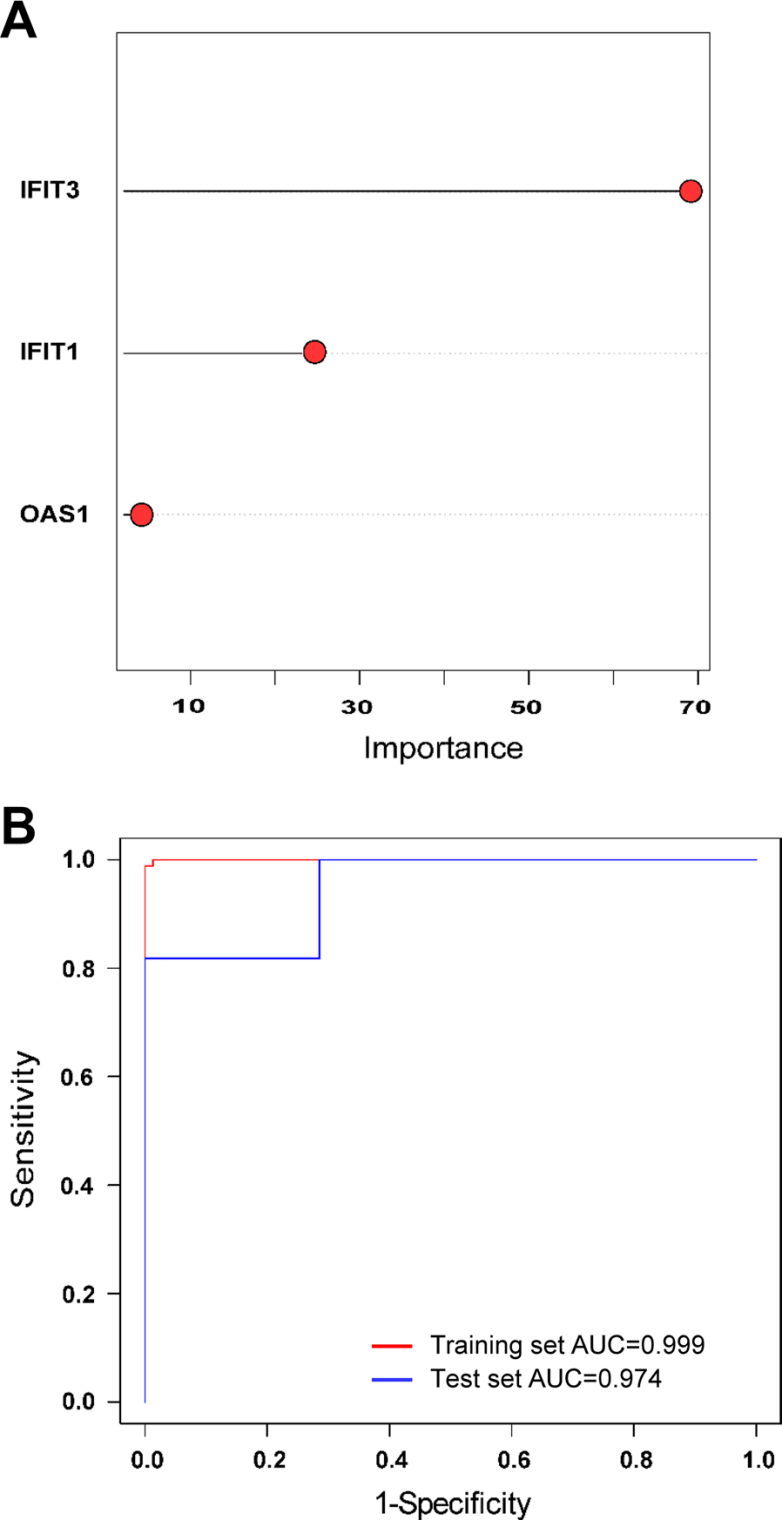
The discriminative performance of the three-gene set in discriminating TB from other diseases based on the Random Forest (RF) predictions in independent GSE37250 dataset. **A** Importance plot of the variables. Total pixel was the three-gene set, followed by *OAS1*, *IFIT1*, and *IFIT3*. **B** ROC of RF prediction model based on the three-gene set in the training set and test set from the GSE37250 dataset. AUC was also shown in the legend area

To quantitatively assess the ability of the three-gene set to classify subjects as having either PTB disease or other diseases, predictive RF models were built based on GSE37250 dataset using 10-cross validation. As a result, the accuracy of this model reached 0.99 in the training set, as well as 0.89 in the test set, showing great predictive performance of RF algorithm. Additionally, we further analyzed the classifier-specific predictive importance of these three gene variables. The number of PTB patients could be predicted in decreasing order of accuracy, when all patients were considered, by *IFIT3* > *IFIT1* > *OAS1* (Fig. 8A). In regards to discriminating PTB from other diseases, AUC values of the three-gene set could reach as high as 0.999 (range 0.999–1) with a sensitivity of 99.0% (98.8–100%) and a specificity of 100% (100–100%) in the training set, and 0.974 (range 0.740–1) with a sensitivity of 96.4% (63.6–100%) and a specificity of 98.6% (85.7–100%) in the test set (Fig. 8B). The high mean AUC, sensitivity, and specificity values (all > 0.90) suggest an overall strong discriminatory ability of this three-gene set between PTB and other diseases, which seems to perform well in the classification of TB and subjects with other diseases within the dataset, despite the different genetic backgrounds of subjects. Overall, these results provide strong evidence that the three-gene set performs well, even in the difficult case of separating PTB from other diseases or healthy controls.

## Discussion

To seek better diagnostics for TB, WHO recently released a consensus target product profile to define the ideal concept of a new diagnostic, which should have a rapid, simple, and low-cost triage test that prioritizes sensitivity to confidently rule out TB, or to identify patients who require further investigation. As not all patients with TB produce sputum spontaneously, a non-sputum confirmatory test that prioritizes specificity is also advocated. Meanwhile, this test should also possess features such as ease of use and reliable detection. Therefore, more attention should be paid to host transcriptional biomarkers that represent a realistic and achievable triage strategy for the resource-limited settings where they are most needed. In this research, in addition to identifying the blood-based signatures (DEGs) in PTB patients compared with HCs, we further explored those biological functions and pathways involved in active PTB. The independent dataset evaluated our bioinformatics results and confirmed that these three genes (*OAS1*, *IFIT1* and *IFIT3*) might potentially be multi-combined diagnostic biomarkers. Finally, the performance of the three-gene set for distinguishing PTB from HCs and patients with other diseases was assessed to explore their potential use as multimarker for PTB diagnostics.

Identifying the immunologic parameters of DEGs involved in active PTB will help us understand the pathogenesis of PTB. The pathogen immune response is a stringently controlled process that must preserve infected organs while eliminating the microbial infection. TB is an immunopathological disease typically affecting the lungs, resulting from an extremely heightened immune response to infection by mycobacteria. Antimicrobial immunity protects the bulk of the infected tissue, but can become harmful when not finely regulated. Active TB as the awful outcome of Mtb infection is based on the interaction between Mtb and host immunity. Troegeler et al. [29] demonstrated that dendritic cell (DC) immunoreceptor sustains type I IFN signaling in DCs, and thus modulates TB immunity in vivo and in vitro. Since DEGs identified were found to be mostly related to type I IFN signaling pathway (Fig. 3), suggesting its high involvement in Mtb infection. Current IGRAs that detect IFN-γ release from T cells in response to two Mtb–specific antigens (ESAT-6 and CFP-10) can determine the presence of Mtb infection based on this theoretical concept [30]. Besides immune response, DEGs were also involved in the virus defense response, and viral genome replication negative regulation using BP (GO) enrichment analysis. These results are consistent with our findings with GSEA analysis and interrelation analysis in immune system pathways for DEGs, as well as BP analysis for hub genes. This observation is further supported by a previous study, in which abortive influenza or hepatitis virus infection in macrophages was found to be controlled through a type I IFN-dependent mechanism [31], suggesting that Mtb infection may share mechanisms with virus infection and viral genome replication.

Additionally, the type I IFN family is a multigene cytokine family that includes one IFNβ, 13 (in humans) or 14 (in mice) partly homologous IFNα subtypes, and a few other gene products [32]. IFNα and IFNβ (IFNα/β) are the most well defined and highly expressed type I IFNs. They are able to stimulate the transcription of a gene program that is able to interfere with the viral replication cycle at multiple stages with numerous mechanisms, and thus mediate the antiviral state in both virus-infected and uninfected cells [33]. Nevertheless, IFNα/β have various accessional roles that regulate both the adaptive and innate immune responses against bacterial and viral pathogens. Both the mouse infection models and patient studies indicate that IFNα/β plays a detrimental role during TB. Several studies have found that the lack of IFNα/β-mediated signaling corresponds to reduced bacterial load and better host survival [34]. Patients suffering from active TB infection have a noticeable whole blood transcriptional profile inducible by IFNα/β, the profiles of which are correlated with the degree of radiographic disease progress, which is reduced after successful treatment [35]. In the absence of the IFNγ pathway, IFNα/β play a part in host protection through experiments in Ifngr1^–/–^Ifnar1^–/–^ mice [36].

Although previous studies have demonstrated the damaging role of type I IFNs during Mtb infection, the mechanism by which IFNα/β exacerbates TB remains unknown. Our KEGG pathway analysis demonstrated that DEGs were largely enriched in RLR signaling pathway (Fig. 3), indicating RLR might involve in the type-I IFN response. Recent studies have reported the distinct roles of the host cell cytosolic RNA sensor RIG-I ((a member of RLR) in the type I IFN signaling in response to Mtb infection [37–39]. Mtb infection induces RIG-I activation, aimed at enhancing downstream signaling. Eira Choudhary et al. further showed that loss of RIG-I in Mtb-infected macrophages leads to dampened IFN-β, IL-1α, and IL-1β production, elevated autophagic activity, and especially reduced intracellular bacterial survival [37]. These results show that targeting this pathway is a potential host-directed approach to treat TB disease. For instant, Shahin Ranjbar et al. firstly clarified that the oral FDA approved thiazolide drug nitazoxanide significantly inhibits intracellular Mtb growth and amplifies Mtb-driven RLR activity [38], further providing robust support that RLR signaling pathway can be further explored in designing novel anti-TB drug targets.

Our study examined the transcriptional profiles of peripheral blood in PTB patients to investigate gene expression changes between PTB and HCs not only based on data analysis, but also qRT-PCR experiments in an independent sample set were used to verify the biological roles of this predictive gene-set in PTB. Finally, *OAS1*, *IFIT1* and *IFIT3* genes were selected as risk indicators of PTB. *2′-5′-OAS*, specifically *OAS1*, *OAS2*, and *OAS3*, are members of IFN-mediated genes that are synonymous with antiviral function. Their upregulation in numerous transcriptome expression profiles, differentiating active and latent infections, was continuously observed through blood transcriptome profiling [40]. This observation is supported by our findings. The upregulated hub genes, such as *OAS1*, *OAS3*, and *OASL*, were identified on the basis of the PPI network of analyzed DEGs. These findings suggested that the hub genes function as clinical biomarkers in PTB. However, the function of *OAS*s and how their expression levels affect the persistence and pathogenesis of TB have yet to be elucidated. The *OAS1* gene is the first of 3 closely related genes located on human chromosome 12q24.2, and encodes three closely linked genes, including *OAS1*, which exist in isoforms p42, p44, p46, and p48 [41]. IFN release induces its expression. As part of the type I IFN response to TB infection, *OAS1* is the most upregulated gene, as shown by neutrophil blood transcriptome profiling in active TB patients [35]. Type I IFN signaling at the late stages of TB is enhanced by the continuous expression of RNase L through *OAS* activation, and *OAS* may exhibit immune-modulatory capabilities [42]. Additionally, considering that the type I IFN response is a significant host response in TB, Type I IFN inducible proteins ISG15, IFIT1, IFIT2, and IFIT3, are highly induced during Mtb infection [43]. The possible role of *ISG15 and IFITs* in the host response to Mtb infection was potentially influenced by type I IFN signaling pathway. While recent studies have reported the distinct roles of *OAS1, IFIT1, and IFIT3* in the type I IFN signaling in response to Mtb infection, a clear and holistic picture of the regulation networks of these three genes involved in host immune during Mtb infection is a complete lack, and other RIG-I-dependent mechanisms involved in Mtb–host interactions are yet to be fully elucidated.

Our results provide strong evidence that the three-gene set could be used to address some of the major challenges in the diagnosis of active PTB. Our three-gene set performed well in the diagnosis of PTB versus HCs, with a mean AUC of 0.911, mean sensitivity of 88.7%, and mean specificity of 91.5% (7 datasets added GSE56153), approximated to the minimum WHO target product profile criteria of 90% sensitivity and 70% specificity for a triage test to rule out TB [44]. In differentiating PTB from other diseases, sensitivity and specificity of RF model with the three-gene set yielded 99% and 100% in the training set, respectively. In the test set, this model provided a sensitivity of 96.4% and a specificity of 98.6%, demonstrating that RF model with the three-gene set had a better power for discrimination between PTB and other diseases. Furthermore, the accuracy of our results is substantially higher than some gene sets from other studies. Pan et al. revealed that the combination of *RETN* and *KLK1* genes achieved the best discriminative capacity (AUC = 0.916), with a sensitivity of 71.2% and a specificity of 93.6%, when discriminating TB from latent tuberculosis infection (LTBI) [45]. However, it failed to reach the WHO target product profile criteria. Recently eight-protein biomarkers were identified using an antibody-based array [46]. Although the training cohort had a 100% specificity and 100% sensitivity in diagnosing TB using the RF algorithm approach by cross-validation, the specificity and sensitivity were 83% and 76% in the test cohort, respectively, as well as 84% and 75% in the prediction cohort. Sweeney et al. [47] showed that the diagnostic power of a set of three genes (*GBP5*, *DUSP3*, and *KLF2*) to separate active TB from HCs with AUC of 0.90, sensitivity of 0.85 and specificity of 0.93, latent tuberculosis with AUC of 0.88, sensitivity of 0.80 and specificity of 0.86, and other diseases with AUC of 0.84, sensitivity of 0.81 and specificity of 0.74 in eight independent datasets composed of both children and adults. Deviations between results could be due to different bioinformatics analysis techniques, such as where the three-gene set was obtained via the calculation of the tuberculosis score. In addition, different selected datasets could contribute to differences in the results. Three datasets (GSE19491, GSE37250, and GSE42834) were used by Sweeney et al. [47] to identify the gene set. Furthermore, differences in biomarker study design might affect the performance of biomarker signatures, as well as the exclusion of HIV-positive individuals and children in our study. Importantly, a principal advantage of the three-gene set is the easy accessibility of blood sampling. However, alternative microbiological tests for TB using non-sputum samples are being developed, which might offer greater promise among patient subgroups where obtaining sputum is difficult. Moreover, the cost of the three-gene set assays is low cost similar to that of C-reactive protein test, which might provide a realistic and achievable diagnostic strategy for the resource-limited settings where they are most needed.

### Limitation

There are some limitations to our study. First, there is insufficient available data to determine whether it is specific to virulent or non-virulent strains, such as *M. tuberculosis* H37Ra or *M. bovis* BCG, since the three-gene signatures were obtained from data based on the multiple clinical PTB cohorts, in which only virulent strains would likely to cause infection, and cells were infected with the virulent strain H37Rv. Second, due to the small number of the samples in independent sample set, the effect of inter-individual differences cannot be avoided, even though independent sample sets were used to evaluate the microarray results. Third, our chosen cohort was restricted to IGRA-negative patients. Similar independent validation studies are needed for LTBI and patients with extrapulmonary tuberculosis. Lastly, due to the lack of publicly available data, the model isn’t specific enough to verify diagnosis alone, but alongside other methods such as differential diagnosis. As well, at the moment there aren’t enough samples to produce a standard that could be quickly referenced. Despite the limitations, the three-gene set, in conjunction with differential diagnosis, can boost the confidence of PTB diagnosis for clinicians, provides an early warning of possible active PTB, and allows for early isolation and treatment, possibly avoiding any potential transmission, and better patient prognosis.

## Conclusion

The data from the 11 dataset cohorts and an independent clinical sample set show that *OAS1*, *IFIT1*, and *IFIT3* were included and evaluated in the multi-combined diagnostic biomarkers in discriminating TB from the control or other disease groups, and were thus regarded as distinctive combined biomarkers of PTB. The commonalities and unique signatures in the blood profiles of the PTB and control samples have considerable implications for PTB biosignature design and future diagnosis, providing insights into the biological processes underlying PTB.

## Supplementary Information

**Additional file 1: Table S1.** Demographic characteristics of the study participants.

**Additional file 2: Table S2.** Primers used in this study.

**Additional file 3: Table S3.** Significantly enriched GO terms and KEGG pathways of DEGs with *P* < 0.05 and gene counts ≥ 3.

## Data Availability

The datasets generated/analyzed for this study can be found in the Gene Expression Omnibus (GSE42834, GSE83456, GSE56153, GSE19491, GSE28623, GSE34608, GSE54992, GSE62525, GSE147964, GSE147690 and GSE37250). Majority of data generated or analyzed during this study are included in this published article and its supplementary information files. Non-included data may be obtained from the corresponding author upon reasonable request.

## Abbreviations

Mtb: *Mycobacterium tuberculosis*
PTB: Pulmonary tuberculosis
HCs: Healthy controls
DEG: Differentially expressed gene
IGRAs: Interferon-γ release assays
GEO: Gene Expression Omnibus
GO: Gene Ontology
KEGG: Kyoto Encyclopedia of Genes and Genomes
BP: Biological process
CC: Cellular component
MF: Molecular function
GTP: Guanosine triphosphate
IFN: Negative interferon
PPI: Protein–protein interaction
PBMCs: Peripheral blood mononuclear cells
DC: Dendritic cell
OAS: Oligoadenylate synthetase
IFIT1: IFN-induced protein with tetratricopeptide repeats 1
RR: Risk ratio
ROC: Receiver operating characteristic
AUC: Area under curve
CI: Confidence interval
SD: Standard deviation
SE: Standard error
