# Supplementary material for: Diagnosis of pulmonary tuberculosis via identification of core genes and pathways utilizing blood transcriptional signatures: a multicohort analysis
Source: Respir Res. 2022 May 14;23:125. doi: 10.1186/s12931-022-02035-4 (PMC9107189; doi:10.1186/s12931-022-02035-4)
Supplement: Supplementary file 1 — Additional file 1: Table S1. Demographic characteristics of the study participants. [file 12931_2022_2035_MOESM1_ESM.doc]

**Additional file 1**

**Table S1 Demographic characteristics of the study participants**

| **Characters** | **PTB (n=20)** | **HCs (n=20)** |
| --- | --- | --- |
| Age, years (median, range) | 31.5 (21-66) | 35 (28-61) |
| BMI (mean ± SD), kg/m^2^ | 21.05 ± 2.6 | 23.23 ± 2.83 |
| Male/female | 9/11 | 8/12 |
| Smokers/non-smokers | 7/13 | 3/17 |
| Previous tuberculosis |  |  |
| Yes/no | 3/17 | 0/20 |
| Sputum smear |  |  |
| Positive | 15 | NA |
| Negative | 5 | NA |
| Sputum culture |  |  |
| Positive | 13 | NA |
| Negative | 2 | NA |
| Not done | 5 | NA |
| Molecular test† |  |  |
| Positive | 15 | NA |
| Negative | 3 | NA |
| Not done | 2 | NA |

Data are n or mean ± SD or median (range). PTB, pulmonary tuberculosis; HCs, healthy controls; n, number of subjects; SD, standard deviation; BMI, body mass index; NA=not applicable. †Molecular tests include Xpert MTB/RIF, RNA and DNA of Mycobacterium tuberculosis were detected.
