# Supplementary material for: Diagnosis of pulmonary tuberculosis via identification of core genes and pathways utilizing blood transcriptional signatures: a multicohort analysis
Source: Respir Res. 2022 May 14;23:125. doi: 10.1186/s12931-022-02035-4 (PMC9107189; doi:10.1186/s12931-022-02035-4)
Supplement: Supplementary file 2 — Additional file 2: Table S2. Primers used in this study. [file 12931_2022_2035_MOESM2_ESM.doc]

**Additional file 2**

**Table S2 Primers used in this study**

| **Primer name** | **Primer sequence 5′ to 3′** |
| --- | --- |
| F_OAS1 | GGTCTATGCTTGGGAGCGAG |
| R_OAS1 | AGTTTCCTGTAGGGTCCGCC |
| F_IFIT1 | GTGCTTGAAGTGGACCCTGA |
| R_IFIT1 | CCTGCCTTAGGGGAAGCAAA |
| F_IFIT3 | TTGGGCAGACTCTCAGATGC |
| R_IFIT3 | TCAAAACACACCTTCGCCCT |
| F_GAPDH | GGAGTCCACTGGCGTCTTCA |
| R_GAPDH | GTCATGAGTCCTTCCACGATACC |
