# Supplementary material for: Diagnosis of pulmonary tuberculosis via identification of core genes and pathways utilizing blood transcriptional signatures: a multicohort analysis
Source: Respir Res. 2022 May 14;23:125. doi: 10.1186/s12931-022-02035-4 (PMC9107189; doi:10.1186/s12931-022-02035-4)
Supplement: Supplementary file 3 — Additional file 3: Table S3. Significantly enriched GO terms and KEGG pathways of DEGs with P < 0.05 and gene counts ≥ 3. [file 12931_2022_2035_MOESM3_ESM.doc]

**Additional file 3**

**Table S3 Significantly enriched GO terms and KEGG pathways of DEGs with *P* < 0.05 and gene counts ≥ 3**

| **Category** | **Term** | **Description** | **Count** | **Count %** | ***P*-value** |
| --- | --- | --- | --- | --- | --- |
| BP term | GO:0060337 | type I interferon signaling pathway | 12 | 19.35483871 | 6.64E-17 |
| BP term | GO:0051607 | defense response to virus | 14 | 22.58064516 | 2.99E-15 |
| BP term | GO:0045071 | negative regulation of viral genome replication | 8 | 12.90322581 | 3.56E-11 |
| BP term | GO:0009615 | response to virus | 9 | 14.51612903 | 1.82E-09 |
| BP term | GO:0060333 | interferon-gamma-mediated signaling pathway | 7 | 11.29032258 | 9.04E-08 |
| BP term | GO:0006955 | immune response | 10 | 16.12903226 | 5.99E-06 |
| BP term | GO:0035456 | response to interferon-beta | 3 | 4.838709677 | 3.47E-04 |
| BP term | GO:0008015 | blood circulation | 3 | 4.838709677 | 0.008872553 |
| BP term | GO:0042981 | regulation of apoptotic process | 4 | 6.451612903 | 0.029638477 |
| CC term | GO:0005739 | mitochondrion | 11 | 17.74193548 | 0.006773073 |
| CC term | GO:0005829 | cytosol | 18 | 29.03225806 | 0.01853107 |
| MF term | GO:0001730 | 2'-5'-oligoadenylate synthetase activity | 3 | 4.838709677 | 5.78E-05 |
| MF term | GO:0003924 | GTPase activity | 5 | 8.064516129 | 0.006194052 |
| MF term | GO:0005515 | protein binding | 38 | 61.29032258 | 0.00645584 |
| MF term | GO:0005525 | GTP binding | 6 | 9.677419355 | 0.006958531 |
| MF term | GO:0003725 | double-stranded RNA binding | 3 | 4.838709677 | 0.015725687 |
| MF term | GO:0016740 | transferase activity | 3 | 4.838709677 | 0.03655713 |
| KEGG | hsa05164 | Influenza A | 4 | 7.142857 | 0.009813 |

GO, Gene Ontology; DEGs, differentially expressed genes; BP, biological process; CC, cellular component; MF, molecular function; GTP, guanosine triphosphate; KEGG, Kyoto Encyclopedia of Genes and Genomes; DEGs, differentially expressed genes.
